# MRI approach to the patient with suspected dementia: artificial intelligence techniques and semi-quantitative rating scales compared

**DOI:** 10.3389/fradi.2026.1667306

**Published:** 2026-01-27

**Authors:** S. F. Calloni, A. Diena, G. M. Agazzi, M. Zavarella, P. Q. Vezzulli, G. Cecchetti, E. G. Spinelli, G. Rugarli, A. Ghirelli, G. Magnani, F. Caso, A. van Loon, F. Agosta, M. Filippi, A. Falini

**Affiliations:** 1 Neuroradiology Unit and Cermac, IRCCS Ospedale San Raffaele, Milan, Italy; 2 Center for Alzheimer’s Disease and Related Disorders (CARD), Neurology Unit, IRCCS San Raffaele Scientific Institute, Milan, Italy; 3 Neurophysiology Service, IRCCS San Raffaele Scientific Institute, Milan, Italy; 4 Vita-Salute San Raffaele University, Milan, Italy; 5 Neuroimaging Research Unit, Division of Neuroscience, IRCCS San Raffaele Scientific Institute, Milan, Italy; 6 Neurology Unit, IRCCS San Raffaele Scientific Institute, Milan, Italy; 7 DeepHealth, Boston, MA, United States; 8 Neurotech Hub, Vita-Salute San Raffaele University, Milan, Italy; 9 Neurorehabilitation Unit, IRCCS San Raffaele Scientific Institute, Milan, Italy

**Keywords:** artificial intelligence, brain volumetry, dementia, magnetic resonance imaging, microbleeds, Quantib ND, visual rating scales

## Abstract

**Objectives:**

To assess the reliability of semi-quantitative and AI-based quantitative brain volume evaluation (Quantib® ND) in predicting clinical diagnosis in patients with suspected neurodegenerative diseases undergoing initial 1.5 T MRI. Additionally, to analyze the frequency of lobar microbleeds (MBs) at diagnosis.

**Methods:**

Two neuroradiologists (2 vs. 10 years’ experience), blinded to diagnosis, independently evaluated brain atrophy on 3D-T1 images of 133 subjects using Scheltens, Koedam, and Kipps scales. Automated volumetric analysis was performed using Quantib® ND. SWI images were assessed by one neuroradiologist to classify MBs as cortical, juxtacortical, subcortical, or deep. Inter-observer agreement was measured using intraclass correlation coefficients (ICC); correlation with Quantib® ND was analyzed using Spearman's coefficient. Cohen's Kappa assessed agreement with clinical diagnosis.

**Results:**

Good inter-observer agreement was observed for the MTA scale (ICC 0.86 right, 0.82 left) and Kipps scale (ICC 0.76), with moderate concordance for Koedam (ICC 0.66). Frontal and posterior temporal Kipps subregions had good concordance (ICC 0.77, 0.79), while anterior temporal showed poor agreement (ICC 0.59). Diagnostic accuracy was moderate across observers and Quantib® ND. Observer 1 showed 77% sensitivity, 51% specificity; observer 2 had 79% sensitivity, 62% specificity; Quantib® ND reached 56% sensitivity, 74% specificity. Patients exhibited significantly more lobar MBs than non-dementia patients (*χ*^2^ *p* = 0.04).

**Conclusions:**

Semi-quantitative visual scales proved effective and sensitive for detecting brain atrophy, showing good concordance with automated volumetric data. While AI-based quantification demonstrated higher specificity, visual assessment remained more sensitive. Lobar MBs were more frequent in neurodegenerative cases.

## 1 Introduction

Dementia affects approximately 55 million people worldwide, with projections indicating that this number could nearly triple by 2050 (1). Alzheimer's disease (AD) and vascular dementia are the leading causes, accounting for around 80% of global dementia cases, with AD responsible for 50%–60% of those (2). As imaging technologies advance, neuroradiology's role in diagnosing cognitive impairment has evolved significantly. What was once primarily focused on excluding diseases is now a crucial tool for clinical diagnosis, subtyping, and ongoing monitoring of neurodegenerative conditions (3). In particular, the role of magnetic resonance imaging (MRI) has expanded as a vital tool for dementia diagnosis. MRI requests are on the rise, especially in centers specializing in neurodegenerative diseases, with the most common field strength being 1.5 Tesla. MRI allows for the assessment of cortical atrophy through visual analysis using semi-quantitative rating scales, which remain highly effective and widely employed in both clinical practice and research for identifying characteristic atrophy patterns in dementia (4). While the quantitative approach is still considered complementary, it promises to enhance the accuracy of diagnosing dementia-related conditions (5, 6). In recent years, artificial intelligence (AI) and machine learning have profoundly advanced neuroimaging-based dementia research. Deep learning architectures—particularly convolutional neural networks (CNNs) and transformer-based models—have achieved high accuracy in detecting and classifying Alzheimer's disease and related dementias from MRI data (7, 8). These algorithms can automatically extract disease-relevant imaging biomarkers from large-scale datasets, often reaching diagnostic performance comparable to expert neuroradiologists.

Nevertheless, these research-oriented AI models remain limited in clinical translation due to dataset heterogeneity, lack of interpretability, and absence of regulatory approval. In contrast, visual rating scales—such as Scheltens' medial temporal atrophy (MTA), Koedam's posterior atrophy, and Kipps' lobar atrophy—are well-established, reproducible tools that remain central to everyday dementia assessment but are inherently observer-dependent. Clinically validated AI-based volumetric software, such as Quantib® ND, offers a practical bridge between these two approaches by providing standardized, quantitative measures of brain atrophy within regulated diagnostic workflows. However, few studies (9) have directly compared the reliability and diagnostic performance of such approved AI tools with traditional visual scales under real-world 1.5 T MRI conditions and across readers with differing levels of expertise—a gap that the present study aims to address.

A key concept in the treatment of neurodegenerative diseases, similar to ischemic stroke, is “time is brain.” Early intervention with disease-modifying therapies for Alzheimer's disease could significantly influence the course of the illness. In this context, integrating the detection of microbleeds (MBs) via susceptibility-weighted imaging (SWI) sequences into the diagnostic process is becoming increasingly important. Microbleeds, particularly when more than four on T2*-GE images, may be linked to adverse side effects (ARIA-A and ARIA-E) in patients receiving newer immunobiological therapies (10).

This study aims to address the following four objectives:

1. Assess the reliability of semi-quantitative evaluation in predicting clinical diagnoses for patients undergoing initial 1.5 Tesla MRI scans with suspected neurodegenerative disease, and compare the performance of two experts.
2. Explore the correlation between semi-quantitative assessments and quantitative cerebral lobar volumetry processed using commercial AI software (Quantib® ND).
3. Evaluate the difference in diagnostic accuracy between semi-quantitative and quantitative methods in detecting cerebral atrophy indicative of dementia.
4. Investigate whether there is a correlation between the number of lobar microbleeds and the clinical diagnosis of dementia.

## 2 Materials and methods

### 2.1 Participants

The local ethics committee for clinical standards on human trials approved this study. Informed consent was obtained from all participants. For this retrospective observational study, patients who underwent MRI with clinical suspicion of cognitive impairment due to a neurodegenerative disease between December 2021 and June 2024 at the Neuroradiology Operating Unit at IRCCS San Raffaele Hospital in Milan, Italy, were included.

Patients were recruited at the Center for Alzheimer's diseases and related disorders, Unit of Neurology and underwent neurological and neuropsychological examinations as part of the diagnostic work-up at the Neurology Unit. Family history was assessed on the basis of direct interview with patients, caregivers and close relatives. A definite clinical diagnosis of neurodegenerative disease was considered as the diagnostic gold standard. Patients receiving a final diagnosis of neurodegenerative dementia at the Memory Clinic were classified according to current international criteria (e.g., NIA–AA criteria for Alzheimer's disease, Rascovsky et al. criteria for behavioral-variant frontotemporal dementia, Gorno-Tempini et al. criteria for primary progressive aphasia variants, and established consensus criteria for other degenerative syndromes, such as posterior cortical atrophy and dementia with Lewy bodies).

The following were considered exclusion criteria for all subjects enrolled in the study: significant comorbidities or substance abuse that could interfere with cognitive functioning; any major/significant systemic, psychiatric, or neurological disease except cognitive impairment; other causes of focal or diffuse brain damage, including lacunes, extensive ischemic findings/major strokes on baseline MRIpresence of significant motion artifacts on routine MRI.

### 2.2 Imaging protocol

The MRI studies were all acquired with a single scanner, Philips d-Stream 1.5 T (Philips Healthcare, Best, The Netherlands), installed at the Neuroradiology Operating Unit. The MRI protocol used in the acquisitions of all subjects included in the study is shown in Supplementary Table S1.

### 2.3 Analysis of MR imaging

#### 2.3.1 Semi-quantitative analysis

MRI images of all subjects were evaluated by two neuroradiologists with mild (observer 1) and high (observer 2) levels of expertise based on their years of neuroradiological experience, (2 and 10 years, respectively).

All 3D T1-weighted images were anonymized for cloud submission to AI brain volumetry quantification software (Quantib® ND).

The two observers performed a semi-quantitative assessment of the degree of atrophy on the 3D-T1 images, in a randomized and independent manner, using Scheltens' (values 0-4) Koedam's (values 0-3) and Kipps' (values 0–4) (8–10) visual rating scales, blinded to the clinical diagnosis (11–13).

- Scheltens scale assessment (MTA) was conducted by considering decade-specific cut-offs by calculating the mean of the two sides, specifically the figure is considered pathological in: subject **<** 65 years if MTA ≥1, subject 65-74 years if MTA ≥1.5, subject 75–84 years MTA ≥2, and subject >85 years if MTA ≥2.
- The Koedam scale assessment was conducted by considering the value of the side with the highest grade, and if this was found to be ≥ 2 then the subject was considered pathological.
- Kipps scale evaluation was conducted by considering among each side and region the highest value, if this was ≥ 2 the patient is considered pathological.

Subjects with a pathological score in at least one of the three scales were classified as pathological MRI patients, while subjects with none of the three positive scales were considered as negative MRI non-dementia patients.

#### 2.3.2 Quantitative analysis

**Figure 1:**
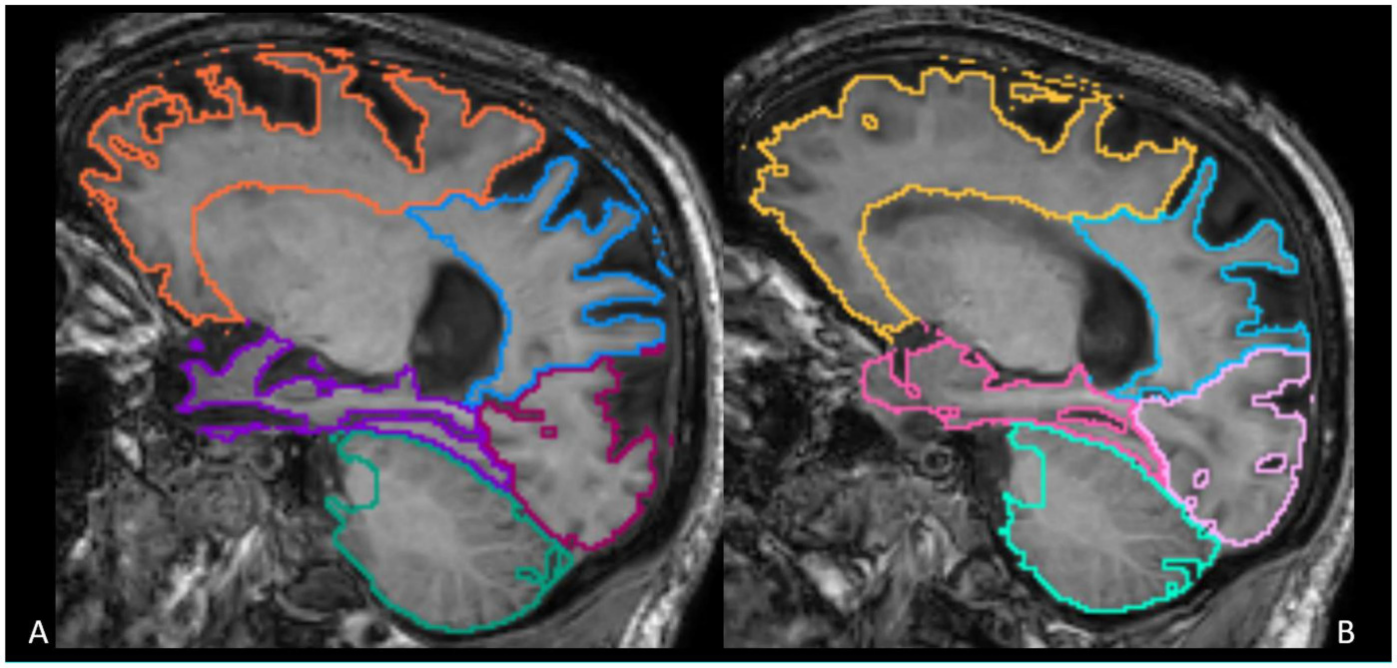
The volumetric segmentation map generated by Quantib® ND with color-coded anatomical regions and corresponding volumetric data both for right **(A)** and left side **(B).**

**Figure 2:**
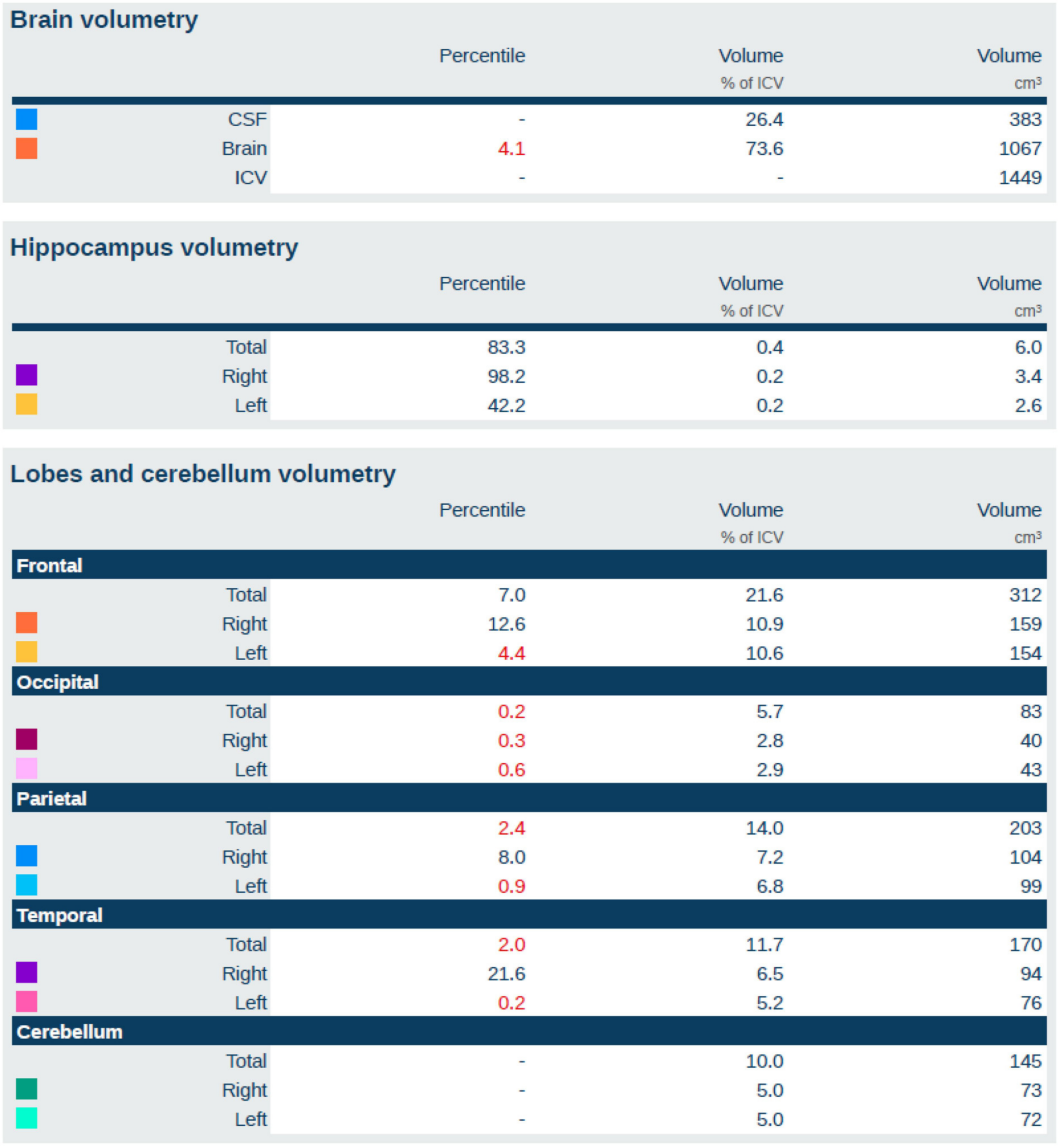
Example of a Quantib® ND atrophy analysis report, illustrating the quantitative percentile rankings of regional brain volumes relative to age- and sex-matched normative data. For each anatomical region, the report displays the percentile (relative to the reference population), the volume expressed as percentage of intracranial volume (ICV), and the absolute volume in cubic centimeters. Shown metrics include: global brain volumetry (top row), hippocampal volumetry (second row), and volumetry of frontal, occipital, parietal, temporal lobes, and cerebellum (subsequent rows). Regions with percentile values below the 5th percentile are automatically highlighted in red.

All 3D-T1 images were de-identified and processed using Quantib® ND software (https://www.quantib.com/solutions/ quantib-nd). Figures 1, 2 show an example of a segmentation of a brain T1 3D weighted image of a dementia patient and the relative report, respectively. This software was chosen because of its relative prevalence in centers dedicated to the study of neurodegenerative diseases, because of its easy clinical applicability, because it is methodologically consistent, and not least because it is FDA and CE approved. The software segments and calculates the volumetry of individual brain lobes divided by side (frontal, parietal, temporal, occipital, and hippocampus) (Figure 1), as well as the total brain volume, identifying volumetry data as pathological if less than the 5 percentile (Figure 2).

### 2.4 Analysis of microbleeds

All magnetic susceptibility imaging (SWI) was anonymized and evaluated by a single neuroradiologist (observer 1). MBs were counted and classified following a topographical criterion and in accordance with literature (14) into superficial (located solely in the gray matter of the cortex) and juxtacortical (at the interface between cortical gray matter and subcortical white matter) and subcortical (located in the white matter without reaching the cortex) and deep MBs according to their relative position to the cortex using coregistered T1-3D images. Cortical, juxtacortical, subcortical MBs were grouped into a single subgroup and divided from deep MBs (14).

Subjects with ≥4 MBs superficial were considered pathological, in accordance with literature data (15) and FDA guidelines about the criteria for inclusion of AD Pz to anti-amyloid Ab drug therapy (16). Literature data have shown that there is an association between the presence of more than 4 microbleeds and cognitive decline. In fact, lobar MBs are associated by a decline in executive function, information processing and mnestic function (14).

### 2.5 Statistical analysis

Differences in categorical variables between groups (patients vs. non-dementia patients) were assessed using the *χ*^2^ test, while differences in continuous variables were evaluated with Student's *t*-test or Wilcoxon's test, depending on distribution normality assessed by the Shapiro–Wilk test (30).

The intraclass correlation coefficient (ICC, two-way random effects, absolute agreement, single measures) was used to assess inter-observer reliability in semi-quantitative ratings. Reliability was interpreted as poor (<0.50), moderate (0.50–0.75), good (0.75–0.90), or excellent (>0.90) (Koo & Li, 2016).

Correlations between quantitative volumetric measures from Quantib® ND and semi-quantitative atrophy scores were analyzed using Spearman's rank correlation coefficient (*ρ*). The MTA score was correlated with hippocampal volume (right and left hippocampus), the Koedam score with mean parietal lobe volume, and the Kipps scale with mean frontal, temporal, and hippocampal lobe volumes. Spearman's coefficient was interpreted as negligible (0 < *ρ* < 0.20), weak (0.21–0.40), moderate (0.41–0.60), strong (0.61–0.80), or very strong (0.81–1.00). Differences in correlations between readers were tested formally (17).

Cohen's Kappa was used to assess agreement in the identification of neuroradiological signs suggestive of neurodegenerative disease between semi-quantitative ratings (for both readers) and Quantib® ND software, using clinical diagnosis as the gold standard. Kappa values were interpreted according to Cohen as follows: 0.01–0.20 (slight), 0.21–0.39 (fair), 0.40–0.59 (moderate), 0.60–0.79 (substantial), and 0.80–1.00 (almost perfect).

Diagnostic performance metrics (sensitivity, specificity, positive predictive value, negative predictive value, and accuracy) were derived from 2 × 2 contingency tables. Ninety-five percent confidence intervals (95% CI) for these metrics were calculated using the Wilson score method. Differences in diagnostic accuracy among readers and Quantib® ND were tested using the method proposed by Roldán-Nofuentes and Sidaty-Regad (2019).

All thresholds applied for semi-quantitative visual scales (Scheltens, Koedam, and Kipps) were pre-specified based on established literature (references 8–10) and not derived from study data. Patients were classified as pathological or non-pathological according to these predefined cut-offs, as described in Section 2.c.

**Figure 3:**
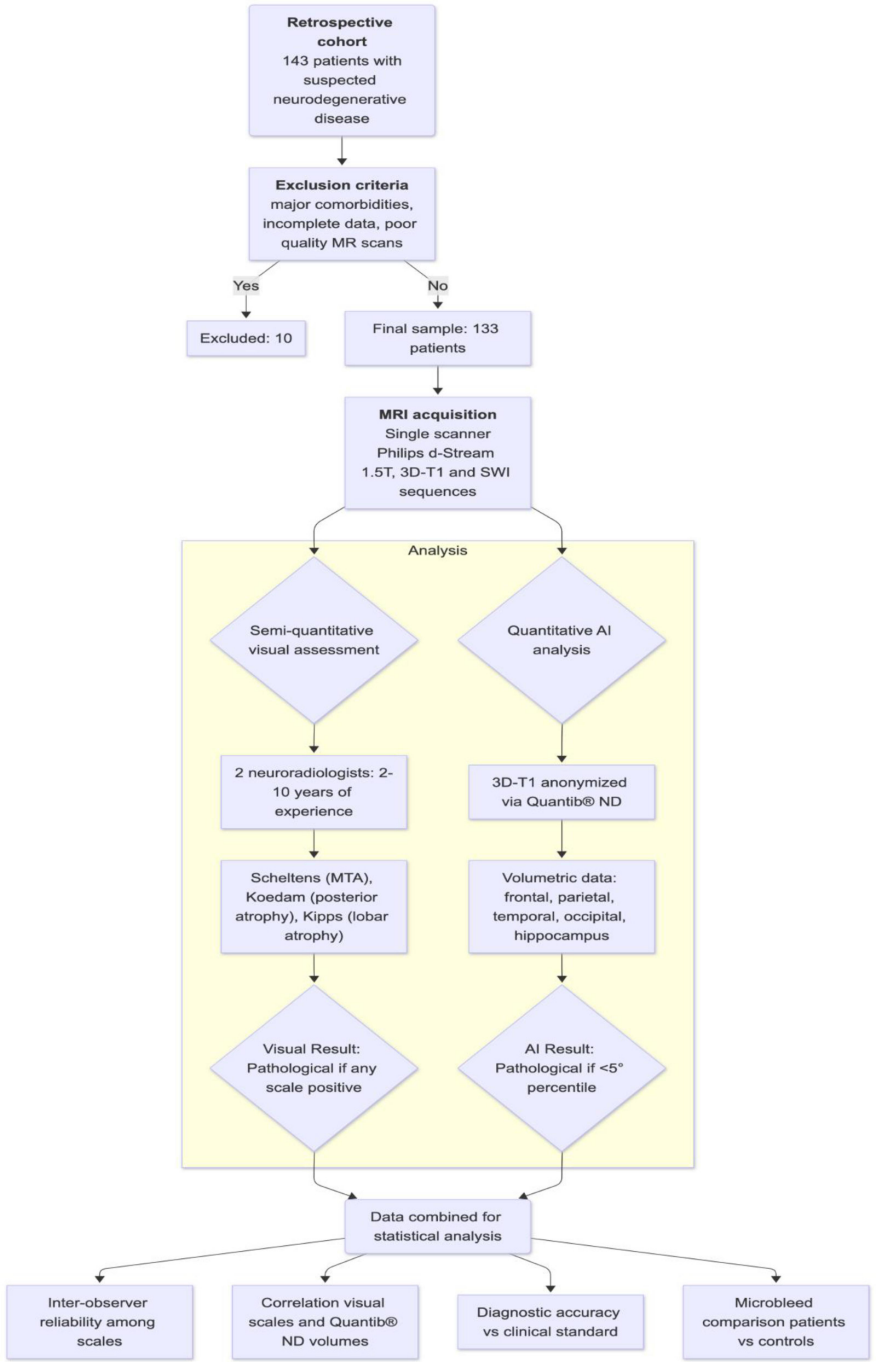
Schematic representation of the study workflow. A total of 133 subjects underwent 1.5 T MRI, including 3D-T1 and SWI sequences. Semi-quantitative visual assessment was performed independently by two neuroradiologists using Scheltens, Koedam, and Kipps scales. Quantitative volumetric analysis was obtained using Quantib® ND software. Microbleeds were classified on SWI images. Statistical analysis included ICC for inter-observer reliability, Spearman's correlation between visual and quantitative measures, Cohen's kappa for diagnostic agreement, and *χ*2 test for microbleed frequency.

Two-sided *p*-values < 0.05 were considered statistically significant. All analyses were performed using R version 4.4.1 (R Foundation for Statistical Computing, Vienna, Austria). Figure 3 graphically shows our study design and analysis pipeline.

## 3 Results

### 3.1 Demographics

143 patients were selected, 10 of whom were excluded due to lack of clinical neurological data (inconclusive diagnoses and/or patients lost to subsequent follow-ups). Thus, a total of 133 patients were included in the study (Supplementary Table S2 shows the different diagnosis distribution).

Patients in the non-dementia group were referred to the neurology unit for subjective complaints or non-specialist clinical evaluation, but specialist clinical assessment, clinical tests, and follow-up did not result in a dementia diagnosis. Non-dementia patients pathological patients did not show statistically significant differences with regard to sex (*p*-value 0.73). However, differences are present with regard to age; in fact, the non-dementia patients were younger relative to the patients (mean age of 66.67 and 73.61 respectively, Mann–Whitney test *p*-value of 0.001), as shown in Table 1.

**Table 1:** Sex and age characteristics of the study cohort.

Demographics	Non-dementia patients	Patients	*p*-value
N	35	98	
Sex (M/F)	16/19	50/48	0.73
Age (years)	66.67 ± 11.18	73.61 ± 8.54	0.002

### 3.2 Concordance between expert and nonexpert observers

Observer 1and Observer 2 showed good concordance for the MTA scale rating for both sides, for the right (ICC 0.86, 95% CI 0.81–0.90) and left (ICC 0.82, 95% CI 0.75–0.87), moderate concordance for the Koedam scale (ICC 0.66, 95% CI 0.55–0.74) and good concordance for the Kipps scale (ICC 0.76, 95% CI 0.70–0.84), respectively. Subclass analysis of Kipps scale shows that observer 1 and 2 have good concordance for frontal region (ICC 0.766 95% CI: 0.685–0.829) and posterior temporal region (ICC 0.791 95% CI: 0.706–0.852) and poor for anterior temporal region (0.589 95% CI: 0.408–0.715), as shown in Table 2.

**Table 2:** Concordance between expert and non-expert observer in the evaluation of the scales used.

Visual scale	Right	Left
MTA	ICC 0.82, 95% CI 0.75–0.87),	(ICC 0.86, 95% CI 0.81–0.90)
Koedam	ICC 0.66, 95% CI 0.55–0.74	
Kipps	ICC 0.76, 95% CI 0.70–0.84	
Frontal Kipps	ICC 0.766 95% CI: 0.685–0.829	
Rear temporal kipps	ICC 0.791 95% CI: 0.706–0.852	
Front temporal kipps	ICC 0.589 95% CI: 0.408–0.715	

### 3.3 Concordance between observers and correlation with volumetry

Spearman correlation values between semi-quantitative assessment and brain volumetry are shown in Table 3.

**Table 3:** Concordance between semi-quantitative assessment and respective brain volumetry.

Visual scale vs. cerebral region	Observer 1 (*ρ*, *p*-value)	Observer 2 (*ρ*, *p*-value)
MTA vs. hippocampus (right)	−0.51, <0.001	−0.60, <0.001
MTA vs. hippocampus (left)	−0.52, <0.001	−0.60, <0.001
Koedam vs. parietal lobe	−0.44, <0.001	−0.46, <0.001
Kipps vs. frontal lobe	−0.38, <0.001	−0.39, 0.007
Kipps vs. temporal lobe	−0.66, <0.001	−0.63, <0.001
Kipps vs. hippocampus	−0.53, <0.001	−0.55, <0.001

### 3.4 Concordance between observers and clinical gold-standards

Concordance between evaluators and clinical gold standard and accuracy metrics obtained from 2 × 2 tables are shown in Table 4.

**Table 4:** Concordance between observer 1, observer 2 and quantib® ND and dementia diagnosis.

Observer/software	Cohen's K (95% CI)	Accuracy	SE	SP	PPV	NPV
Observer 1	0.28 (0.1–0.45)	0.71 (0.62, 0.78)	0.77 (0.68, 0.85)	0.51 (0.34, 0.69)	0.82 (0.72, 0.89)	0.45 (0.29, 0.62)
Observer 2	0.39 (0.21–0.56)	0.75 (0.67, 0.82)	0.79 (0.70, 0.87)	0.62 (0.44, 0.78)	0.86 (0.77, 0.92)	0.51 (0.35, 0.67)
Quantib® ND	0.23 (0.08–0.37)	0.61 (0.52, 0.69)	0.56 (0.46, 0.66)	0.74 (0.56, 0.87)	0.86 (0.75, 0.93)	0.37 (0.26, 0.50)

Sensitivity (Se); Specificity (Sp); positive predictive value (PPV); negative predictive value (NPV).

Observer 2 obtained higher accuracy and better Cohen's K than the Quantib® ND software and the inexperienced evaluator. The differences in Cohen's K were not statistically significant because of the overlapping 95% CI between the three groups. The difference in diagnostic accuracy of inexperienced vs. experienced reader is not statistically significant (*p*-value >0.05). The difference in diagnostic accuracy of Quantib® ND vs. observer 1 and 2 are statistically significant (*p*-value <0.001).

The assessment performed independently by the two observers with semi-quantitative scales showed greater sensitivity than the Quantib® ND assessment, which showed greater specificity.

### 3.5 Differences in microbleeds

Microbleeds were assessed on SWI and classified according to their anatomical location as lobar (cortical, juxtacortical, or subcortical) or deep (basal ganglia, thalamus, brainstem, or cerebellum). In the final dataset (*n* = 137), microbleed prevalence differed substantially between diagnostic groups (*χ*2 *p*-value 0.04). Among patients with dementia (*n* = 98), 44 subjects (44.9%) exhibited at least one lobar microbleed, whereas this was observed in only 2 out of 39 non-dementia patients (5.1%). Deep microbleeds were also more frequent in the dementia group (17/98; 17.3%) than in the non-dementia patients group (2/39; 5.1%). Regarding burden, ≥4 lobar microbleeds were identified in 15 dementia patients (15.3%) and in only one non-dementia subject (2.6%).

## 4 Discussion

Magnetic resonance imaging (MRI) has long been an essential tool in diagnosing suspected dementia, not only for ruling out secondary causes but also for targeting clinical diagnoses by documenting specific brain regions and patterns of atrophy. Unlike many studies that evaluate dementia patients based on solid clinical suspicion in a research setting (6, 18–20), our study mirrors everyday clinical practice, where patients often present without a precise clinical framework. This is in addition to the absence of laboratory data or nuclear medicine imaging to corroborate the neuroradiological findings. In this context, we demonstrate how both semi-quantitative rating scales and brain volumetry processed by artificial intelligence (AI) software consistently contribute to the diagnostic process for patients with suspected dementia. Our analysis benefits from a homogeneous dataset, as all patients underwent the same MRI protocol on the same 1.5 Tesla scanner—considered the gold standard in clinical practice and the most widely used MRI magnet model in the region. This robust design strengthens our findings, ensuring consistency across the data. The first analysis compared the results of two evaluators with varying expertise in using semi-quantitative rating scales (11–13). We employed three distinct scales (Medial Temporal Lobe - MTA, Koedam, and Kipps) to evaluate different neurodegenerative disorders (Alzheimer's, Posterior Cortical Atrophy -PCA, and Frontotemporal dementia -FTD), a necessity since our patients lacked major diagnostic suspicion or specific biomarkers. The standard neuroradiological approach was followed to assess atrophy location and severity. The final diagnosis, made through comprehensive clinical, laboratory, and instrumental evaluation, served as the gold standard.

Our results align with existing literature, showing that both raters had a strong agreement in semi-quantitative assessments (4, 18). Notably, the highest agreement was observed using the Kipps scale, followed by the Schelten scale. This may be because these scales more directly capture regions vulnerable to dementia pathology, where changes are more readily identifiable. When examining the subdomains of the Kipps scale, we found good concordance in the frontal and posterior temporal regions, but lower agreement in the anterior temporal region. This discrepancy likely arises from the anatomical challenges in assessing the anterior temporal area, which lacks clear landmarks compared to the more easily identifiable posterior temporal region. In our memory-clinic cohort, the majority of patients had a clinical diagnosis of Alzheimer's disease. The Scheltens MTA and Kipps lobar atrophy scales predominantly target medial temporal and frontal/temporal regions, which are commonly and relatively early involved across these disorders, particularly in typical late-onset Alzheimer's disease and behavioral-variant frontotemporal dementia. This may, at least in part, explain the higher agreement we observed for these scales compared with the Koedam posterior atrophy scale. Nevertheless, we acknowledge that different dementia subtypes exhibit distinct atrophy patterns, and our mixed cohort does not allow a subtype-specific validation of each scale. We have therefore rephrased our conclusions to emphasize that our findings relate to a heterogeneous, real-world memory-clinic population rather than to any single nosological entity.

Regarding the Koedam scale, we found moderate concordance, likely due to its requirement to assess multiple cortical areas across several planes, which may complicate the agreement between raters on subtle atrophic changes (21). Additionally, the less experienced observer tended to overestimate findings, a pattern that underscores the importance of training in using these scales in clinical practice.

In our second analysis, we found moderate diagnostic accuracy between the scores generated by the two observers and the brain volumetry data processed by Quantib® ND. This validates the role of semi-quantitative scales in the diagnostic approach, as they help define site-specific atrophy and gauge severity, aligning with the findings of several published studies (4, 18, 20, 22). Interestingly, correlation between semi-quantitative ratings and volumetry was strongest for the hippocampal and posterior temporal regions, as seen with the MTA and Kipps scales. In contrast, correlation was weaker for the parietal and frontal lobes, where the Koedam and Kipps frontal subdomains showed lower agreement. This aligns with literature suggesting that Alzheimer's disease, especially in late-onset forms, primarily affects the temporo-mesial regions (23).

The third analysis examined how well semi-quantitative assessments and brain volumetry software could correlate with and predict a clinical dementia diagnosis. Our findings highlighted a difference between the more experienced and less experienced observers, emphasizing the need for adequate training in using these visual scales (4, 6, 21). Notably, visual rating scales demonstrated greater diagnostic sensitivity than brain volumetry alone, though the latter showed higher specificity. This suggests that volumetric data should not be relied upon in isolation; rather, it must be interpreted alongside semi-quantitative grading. The greater sensitivity of the semi-quantitative scales aids in capturing a broader range of dementia cases, albeit at the cost of increased false positives, whereas brain volumetry's specificity helps exclude definitively non-pathological cases.

These results highlight the potential synergy between AI-based brain volumetry and visual rating scales in diagnosing cognitive impairment. Specifically, using volumetric data to rule out non-pathological cases could prevent unnecessary follow-up exams, reduce healthcare costs, and streamline patient management. Previous studies have shown that combining AI and semi-quantitative scales improves diagnostic accuracy and reduces false positives, especially for Alzheimer's and FTD patients, and enhances the diagnostic performance of less experienced readers (18, 23, 24). While some studies question the clinical utility of AI in this context (25–27), we believe the contradictory results may stem from inconsistent normative data and non-standardized imaging protocols. To address this, our study used a consistent MRI protocol with FDA and CE-approved software, ensuring robust, reproducible results.

Our final analysis examined the presence of microbleeds (MBs), revealing a correlation between lobar MBs and a clinical dementia diagnosis. Lobar microbleeds (MBs) play an increasingly important role in the differential diagnosis and management of patients with cognitive impairment. Their presence can help distinguish neurodegenerative disease–related atrophy from vascular or amyloid angiopathy–related pathology, particularly when MBs are predominantly cortical or juxtacortical. This distinction is clinically relevant, as extensive lobar MBs are often associated with cerebral amyloid angiopathy and may contraindicate or complicate anti-amyloid monoclonal antibody therapy due to the risk of amyloid-related imaging abnormalities (ARIA-A and ARIA-E). Consistent with previous studies (28), our findings support the inclusion of MB assessment as a complementary imaging biomarker in the diagnostic work-up of dementia, contributing both to etiologic differentiation and to safer therapeutic decision-making (16, 18).

Although AI-based volumetric tools such as Quantib® ND currently serve as complementary aids rather than replacements for expert visual assessment, they hold considerable potential to improve diagnostic workflow in the near future. By providing standardized, automated quantification of regional brain atrophy, these systems can significantly reduce reporting time, increase consistency across readers, and support faster clinical decision-making, particularly in high-volume or general radiology settings. In addition, the integration of such tools into routine practice may streamline diagnostic pathways and ultimately lower healthcare costs by reducing the need for repeated or ancillary examinations. Future cost-effectiveness and workflow studies are warranted to confirm these anticipated advantages and to define the optimal balance between automated analysis and expert interpretation. Looking forward, it would be valuable to replicate this analysis in a similar cohort using a 3 T MRI scanner and conduct follow-up MRIs to track how quantitative and semi-quantitative data evolve over time, potentially identifying early radiological signs of dementia.

A key limitation of our study is the absence of a community-based healthy control group; instead, our comparator group consisted of patients referred to the memory clinic with cognitive complaints or subjective memory concerns in whom a dementia diagnosis was ultimately ruled out after specialist assessment and follow-up. Also, the exclusion of certain semi-quantitative scales like GCA (29)or those more sensitive, such as those described by Harper et al. (4). We also did not correlate scale scores with specific dementia patterns but rather with a broad clinical diagnosis of dementia. The proportion of dementia vs. non-dementia cases reflects the case-mix of a tertiary memory clinic and resulted in unbalanced group sizes. Although this does not invalidate the calculation of diagnostic performance metrics derived from 2 × 2 contingency tables, it may limit generalizability to settings with a different prevalence of dementia. Additionally, we did not conduct follow-up MRIs, which could confirm or refine our initial findings and track the progression of atrophy. Lastly, the Kipps scale, which separates the anterior and posterior temporal lobes, may not fully align with volumetric analyses that assess the entire temporal lobe, introducing potential discrepancies in the findings. Microbleed assessment was performed by a single neuroradiologist, which prevented evaluation of inter-rater reliability and may have introduced observer-related bias in MB detection. Although our findings demonstrate good agreement between imaging-based evaluations and clinical diagnosis, a key limitation of this study is the absence of correlation with neuropsychological performance, disease severity scales, or biological markers such as cerebrospinal fluid (CSF) and positron emission tomography (PET) data. These parameters could have provided additional validation of the imaging results and strengthened the clinical relevance of our conclusions. Future prospective studies combining MRI with biomarker and cognitive assessments are warranted to establish a more comprehensive diagnostic model that integrates structural, functional, and biochemical measures of neurodegeneration.

## 5 Conclusions

Our study thus validated the role of semi-quantitative rating scales as an effective and sensitive tool in the neuroradiological diagnostic approach to the patient with suspected dementia. The use of artificial intelligence software to quantify the brain atrophy finding is also an important supplementary element to the diagnostic work-up as it ensures greater homogeneity of results when used by observers with different degrees of experience, especially allowing the exclusion of pictures that are not definitely pathological to the imaging finding.

## Data Availability

The datasets presented in this article are not readily available. Requests to access the datasets should be directed to calloni.sonia@hsr.it.
