# Supplementary material for: MRI approach to the patient with suspected dementia: artificial intelligence techniques and semi-quantitative rating scales compared
Source: Front Radiol. 2026 Jan 27;6:1667306. doi: 10.3389/fradi.2026.1667306 (PMC12887851; doi:10.3389/fradi.2026.1667306)
Supplement: Supplementary file 1 [file Supplementaryfile1.docx]

**Supplementary Materials**

**Table 1:** *Table 1: Imaging protocol performed with Philips d-Stream 1.5T scanner (Philips Healthcare, Best, The Netherlands)*

*
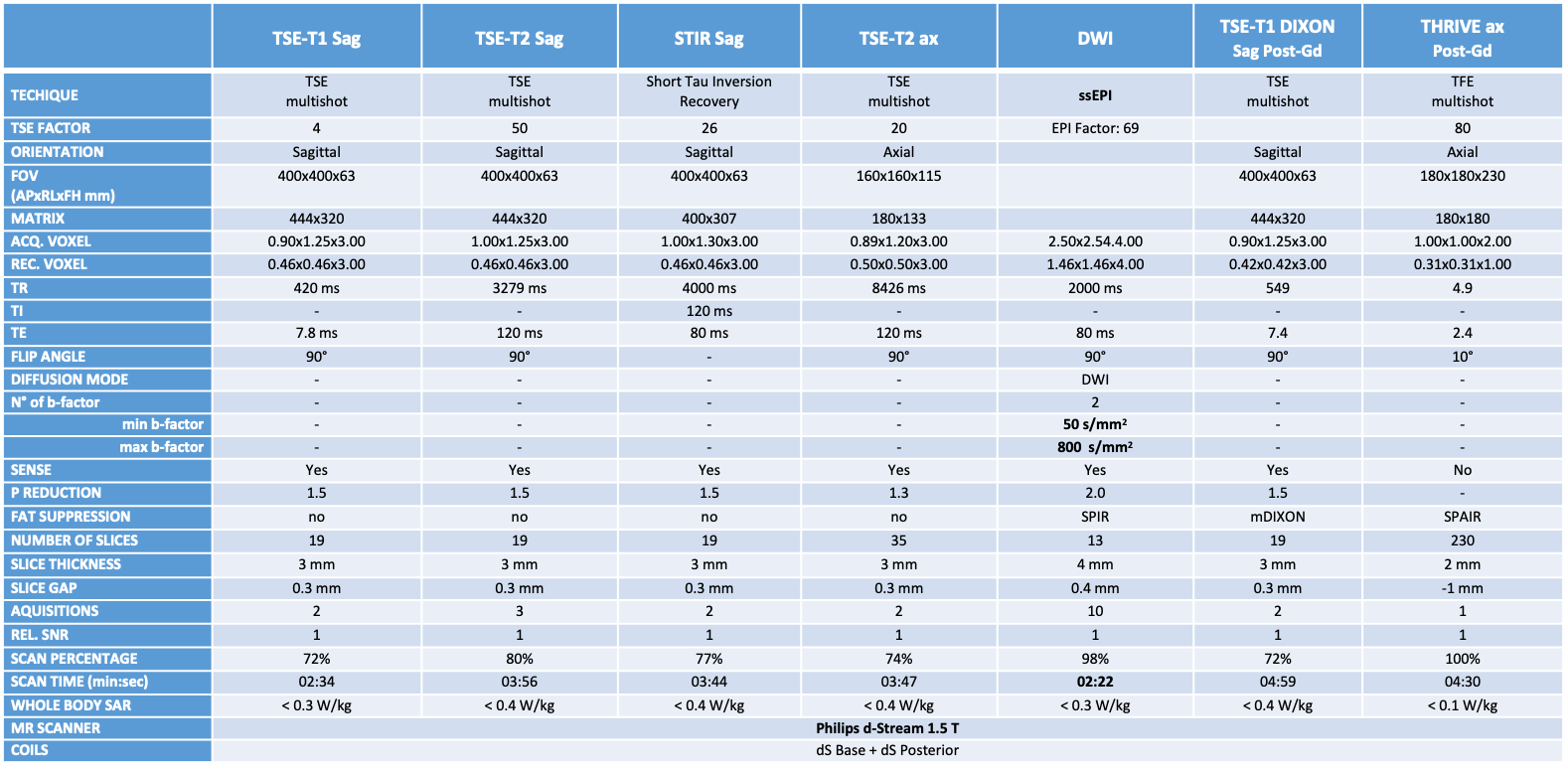
*

**Table 2:** *Distribution of final clinical diagnostic categories in the cohort. Diagnostic labels from the clinical records were harmonized into aggregated categories according to established nosological groups (e.g., AD, VaD, FTD, PD-spectrum, atypical parkinsonism)*

| Diagnostic Category | Included Labels | N |
| --- | --- | --- |
| Alzheimer’s disease (AD) | AD; Probabile AD; Sospetto AD | 25 |
| AD + Vascular / Mixed-AD | AD + VaD; AD + VaD e segni di parkinsonismo | 14 |
| Vascular dementia (VaD / VCI) | VaD; Vad; VCI | 11 |
| Mixed dementia (uncertain mix) | Mixed; Mixed? | 10 |
| Parkinson’s disease / Parkinsonismi | PD; Parkinsonismo; Parkinsonismo ; PD vs iatrogeno/funzionale; Sospetto parkinsonismo atipico | 10 |
| Frontotemporal dementia (FTD) | sbvFTD; bvFTD?? | 2 |
| Dementia with Lewy bodies (LBD) | LBD | 1 |
| Normal Pressure Hydrocephalus (NPH) | NPH | 7 |
| Progressive Supranuclear Palsy (PSP) | PSP | 2 |
| CAA (possible cerebral amyloid angiopathy) | CAA | 1 |
| Inconclusive / Not specified | Not Specified; Not specified; Inconclusive | 5 |
